# Toward a Wireless Open Source Instrument: Functional Near-infrared Spectroscopy in Mobile Neuroergonomics and BCI Applications

**DOI:** 10.3389/fnhum.2015.00617

**Published:** 2015-11-12

**Authors:** Alexander von Lühmann, Christian Herff, Dominic Heger, Tanja Schultz

**Affiliations:** ^1^Machine Learning Department, Computer Science, Technische Universität BerlinBerlin, Germany; ^2^Institute of Biomedical Engineering, Karlsruhe Institute of TechnologyKarlsruhe, Germany; ^3^Cognitive Systems Lab, Karlsruhe Institute of TechnologyKarlsruhe, Germany

**Keywords:** open source, functional near-infrared spectroscopy (fNIRS), brain computer interface (BCI), modularity, wearable devices, neuroergonomics

## Abstract

Brain-Computer Interfaces (BCIs) and neuroergonomics research have high requirements regarding robustness and mobility. Additionally, fast applicability and customization are desired. Functional Near-Infrared Spectroscopy (fNIRS) is an increasingly established technology with a potential to satisfy these conditions. EEG acquisition technology, currently one of the main modalities used for mobile brain activity assessment, is widely spread and open for access and thus easily customizable. fNIRS technology on the other hand has either to be bought as a predefined commercial solution or developed from scratch using published literature. To help reducing time and effort of future custom designs for research purposes, we present our approach toward an open source multichannel stand-alone fNIRS instrument for mobile NIRS-based neuroimaging, neuroergonomics and BCI/BMI applications. The instrument is low-cost, miniaturized, wireless and modular and openly documented on www.opennirs.org. It provides features such as scalable channel number, configurable regulated light intensities, programmable gain and lock-in amplification. In this paper, the system concept, hardware, software and mechanical implementation of the lightweight stand-alone instrument are presented and the evaluation and verification results of the instrument's hardware and physiological fNIRS functionality are described. Its capability to measure brain activity is demonstrated by qualitative signal assessments and a quantitative mental arithmetic based BCI study with 12 subjects.

## 1. Introduction

Functional Near-Infrared Spectroscopy (fNIRS) is an increasingly established technology pioneered by Jöbsis (1977) that allows non-invasive, comparatively low-cost, compact and hazard-free continuous measurement of cerebral oxygenation levels using near-infrared light.

While first generation instruments were rather bulky and expensive, using Laser Diodes with Photo Multiplier Tubes (PMTs) (Cope and Delpy, 1988; Cope, 1991; Rolfe, 2000; Schmidt et al., 2000) and later Avalanche Photo Diodes (APDs) (Boas et al., 2001; Coyle et al., 2004, 2007), today's devices often take advantage of Light Emitting Diodes (LED) and Photo Diodes (PDs) (Vaithianathan et al., 2004; Bunce et al., 2006; Chenier and Sawan, 2007; Ayaz et al., 2013; Safaie et al., 2013; Piper et al., 2014) which allow safe, more compact and mobile applications. After the initial development of laboratory and bedside-monitoring devices for monitoring of local oxygenation levels e.g., in newborn infants (Cope and Delpy, 1988; Cope, 1991), in the 2000s many research groups focused on the design of imaging instruments for brain activity mapping from topographic information [functional Near-Infrared Imaging (fNIRI)] (Schmidt et al., 2000; Boas et al., 2001; Vaithianathan et al., 2004). Recently, fNIRS and fNIRI have entered neuroscience as a reliable and trustworthy research tool for research based on investigating groups of subjects (Scholkmann et al., 2014), offering potentially complementary information to fMRI, PET and EEG (e.g., oxygenation information or cytochrome oxidase as marker of metabolic demands; Strangman et al., 2002). But also in adjacent fields such as Brain Computer Interfaces (BCI) and neuroergonomics, defined as the study of the human brain in relation to performance at work, (Parasuraman, 2003, 2011), fNIRS technology opens new possibilities (see e.g., Matthews et al., 2008, for an introduction in hemodynamics for Brain-Computer Interfaces). It is increasingly built for and used in single-trial fNIRS applications for BCIs for control (Naseer and Hong, 2013; Schudlo and Chau, 2015) and rehabilitation (Kanoh et al., 2009; Yanagisawa et al., 2010) and has successfully been used for cognitive workload assessment (Son and Yazici, 2006; Ayaz et al., 2012), brain dynamics monitoring during working memory training and expertise development (Ayaz et al., 2013), hybrid NIRS-EEG based signal processing tasks (Safaie et al., 2013; Putze et al., 2014) and recently also in combination with trans-cranial direct current stimulation (tDCS; McKendrick et al., 2015). Furthermore, fNIRS is found to be a promising multimodal expansion to EEG-based BCI (Pfurtscheller et al., 2010; Biessmann et al., 2011; Fazli et al., 2012). The major limitation of fNIRS is the relatively slow onset of hemodynamic processes. However, especially in the field of passive BCI (Zander and Kothe, 2011), reaction times do not necessarily have to be extremely fast.

An increasing number of approaches using fNIRS in the field of mobile brain imaging (e.g., Piper et al., 2014) and neuroergonomics (Fairclough, 2009) shows the demand for wireless, miniaturized and customizable fNIRS technology. So far, researchers either relied on costly and mainly static commercial devices, or designed their own fNIRS equipment from scratch. Trying to overcome the restrictions of commercial tabletop instruments, the latter was done by groups such as Ayaz et al. (2013), Safaie et al. (2013), Lareau et al. (2011) and Atsumori et al. (2007), using LEDs with Si PDs/APDs for new generation instruments that generally enable a mobile use. To the best of our knowledge however, only very few of even newer generation devices (Safaie et al., 2013) are truly miniaturized, stand-alone, unobtrusive and mobile and can be carried on the body without a backpack while still enabling free movement and data transmission/processing at the same time, as often external static instrumentation such as DAQ-equipment, lock-in amplifiers and power sources are required. Also, in many cases, signal extraction technologies like lock-in amplification seem to be sacrificed for the sake of miniaturization or complexity. Probe and attachment designs proposed in the last years, such as the use of flexible PCBs (Vaithianathan et al., 2004; Bozkurt et al., 2005; Bunce et al., 2006; Son and Yazici, 2006; Rajkumar et al., 2012), eeg-cap like optodes (Kiguchi et al., 2012; Piper et al., 2014) and mechanical mounting structures (Coyle et al., 2007) are usually limited to static applications and/or in case of flexible PCB to a fixation on the forehead due to obstruction by hair. An often reported issue in the field of mobile applications, that seems not to be resolved satisfactorily so far, is the optode attachment to the head for both stable optical contact, sufficient light levels and comfortable wearing.

While the recent trend of new system designs for portability and mobility can also increasingly be observed in commercial devices, researchers will always need custom solutions for innovative approaches. To help reducing the time and effort in these cases, we present the design and a first evaluation of a configurable, miniaturized, modular, fully mobile (wireless) multichannel fNIRS system that is provided open source on www.opennirs.org under a CC BY-NC 4.0 license with a detailed documentation. It is a customizable low-cost research tool that enables both stand-alone use and the combination with custom or external DAQ equipment. Also, the device makes use of a new detailed spring-loaded optode fixation concept to tackle the above mentioned optode attachment issue.

## 2. Materials and methods

### 2.1. Instrument requirements

We identified aspects that are crucial to be fulfilled for a fNIRS device in the context of mobile BCI and neuroergonomics. Besides the criterion for the hardware to be comparatively low cost, these can be assigned to four groups:

- **Usability:** Miniaturization and mobility of the device, unobtrusiveness and robustness of the optode attachment.
- **Signal Quality:** Low inter channel crosstalk, low drifts of light sources and overall system signals, high signal sensitivity/amplifier precision, robustness to background light and high dynamic range.
- **Safety:** Low heat development, harmless light intensities and galvanic isolation to power lines.
- **Configuration/Customization:** Scalability of channel number, modularity, configuration of light intensities and receiver gain, interface to custom hard-/software.

The following subsections will provide detailed information on our approach to fulfill these requirements on a concept, hardware and software level.

### 2.2. Instrumentation design

#### 2.2.1. System concept

**Figure 1:**
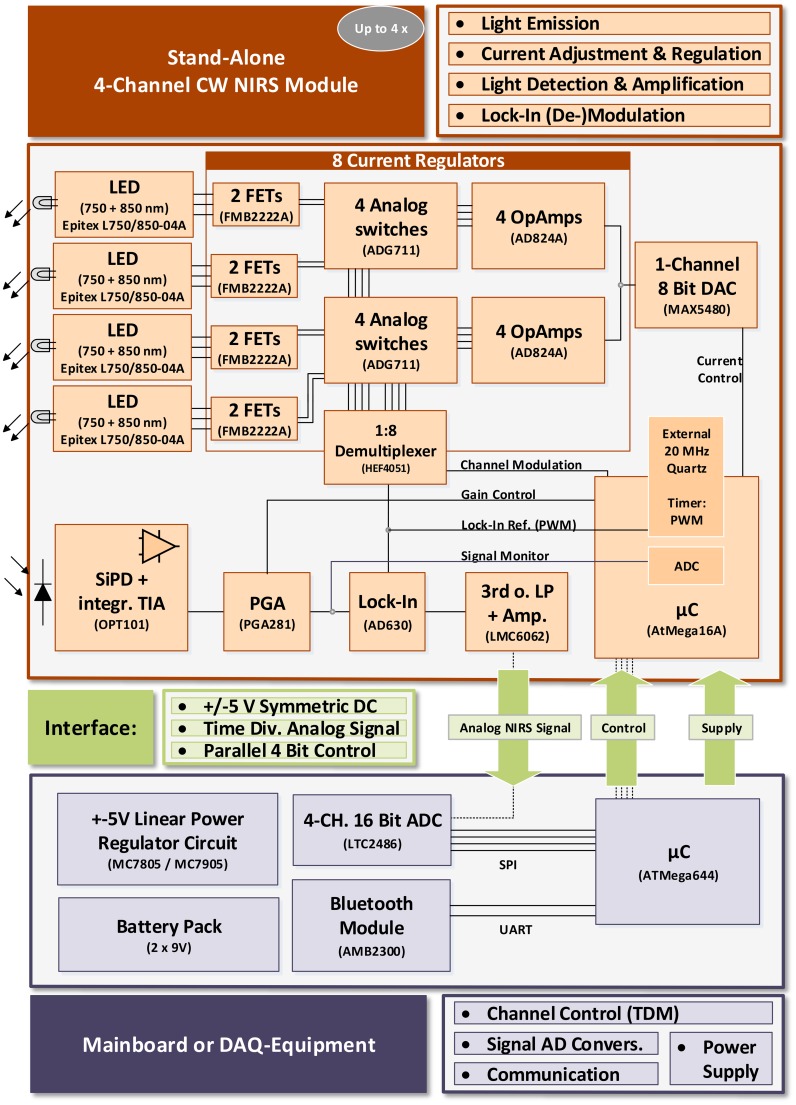
**System Concept**.

The system concept of the modular open instrument is shown in Figure 1. It consists of one or more stand alone 4-channel Continuous Wave NIRS modules and a mainboard. Each module is controlled by the mainboard via a simple parallel 4 Bit control interface. The mainboard provides the power supply rails, AD-conversion of the NIRS signals and an UART communication interface and can be replaced by any custom data acquisition (DAQ-) equipment when the control interface and symmetric ±5 V power rail are supplied. This enables full customization of the instrument with respect to physical channel number, power consumption and conversion rate and depth, while spatially distributing the hardware components (and weight), and performing local hardware signal amplification and processing, thus minimizing noise and interferences.

The fNIRS modules were designed considering the current understanding of fNIRS instrumentation technology as reviewed by Scholkmann et al. (2014) and others (Obrig and Villringer, 2003; Son and Yazici, 2006) with special regard to hardware design and wavelength-selection for SNR maximization/crosstalk minimization and considering potential hazards as identified by Bozkurt and Onaral (2004).

Each module provides four dual wavelength fNIRS channels using 750 and 850 nm multi-wavelength *Epitex L750/850-04A* LEDs. While the LEDs have a broader emission spectrum (Δλ = 30/35 nm) than sharp peaked laser diodes (typically Δλ≈ 1 nm), their incoherent and uncollimated light allows for a higher tissue interrogation intensity and direct contact with the scalp due to less heating and is safer with the human eyes.

The LED current is regulated by adjustable current regulator circuits based on high precision amplifiers (*Analog Devices AD824A*) and field effect transistors (FMB2222A). Channel activation and current modulation for lock-in amplification is performed by analog switches (*Analog Devices ADG711*) that are accessed via an analog 1:8 demultiplexer (*NXP HEF4051*). After tissue interrogation, NIR light is detected by a central Si photo detector with integrated trans-impedance amplifier for output noise minimization (*Texas Instruments OPT101*, 1 MΩ feedback resistor, bandwidth 14 kHz) and is then amplified and lock-in demodulated (using Analog Devices AD630). An 8 Bit *Atmel Corp. AtMega16A* microcontroller's PWM module creates the 3.125 kHz square wave reference for lock-in (de-)modulation using an external 20 MHz crystal for jitter minimization. It also processes incoming control signals from the 4 Bit control interface and operates and configures the on board hardware. For adjustment of the LED currents, an 8 Bit digital-to-analog converter (DAC; *Maxim MAX5480*) is implemented. It supplies the voltage level at the current regulator inputs that is the command variable for the current regulation level. A programmable gain amplifier (*Texas Instruments PGA281*) is implemented for pre lock-in amplification of the detected NIR signal with a variable gain from *G* = 0.688 to 88.

During lock-in demodulation, the signal is filtered by a 3rd-order Butterworth low-pass and is then again amplified (*G* = 5.1) and stabilized by a set of two high precision amplifiers (*Texas Instruments LMC6062*) before leaving the fNIRS module for external AD conversion.

The system is designed for Time-Division Multiplexing (TDM) of the fNIRS channels. This is a trade-off between minimizing inter-channel crosstalk, heating (Bozkurt and Onaral, 2004) and battery consumption on the one hand and sacrificing SNR, which is limited by the width of the applied time windows. For demultiplexing of the locked-in output branches, a variable (sample rate dependent) dwell time is inserted after each onset of a single channel activation before sampling the steady state photo detector signal on the mainboard or with custom DAQ equipment.

Configurable PGA gain (*G* = 0.6875–88) and LED-intensity (256 DAC levels) in combination with a feedback “signal monitor” line allow the signal dependent adaption for maximum amplification in the lock-in demodulation process without reaching the dynamic range limit of one of the components.

***Modularity:*** The above described design of the fNIRS modules allows operation in many configurations—only requiring compatibility with the above mentioned interface consisting of 4 Bit control, power supply and analog output. For an extension of the total channel count, several modules can be used. Changes in set-up and module count only affect the control unit and its routines chosen by the user, which activate the time division multiplexed channels and convert the analog fNIRS signals from the modules:

- As the objective of this work was the design of miniaturized fNIRS modules for mobile applications, a microcontroller (*Atmel AtMega644*) based mainboard was developed for mobile data acquisition and module control. Using a 4 channel 16 Bit analog-to-digital converter (ADC; *Linear Technologies LTC2486*) and a Bluetooth wireless controller (*Amber Wireless AMB2300*), the mainboard acquires the fNIRS signal(s) from up to 4 modules (16 channels), transmits the data to a computer via serial protocol and processes incoming user controls. To scale the number of channels, the user connects the desired number of modules and configures the channel administrator routine on the mainboard's microcontroller (see also Section 2.2.3). The symmetric ±5 V power rail is created from battery DC voltage using a stabilized linear power regulator circuit (based on *ON Semiconductor MC7805 and MC7905* ICs). Running on batteries and using only low voltages also ensures user safety.
- The mainboard is a placeholder for any (custom) peripheral acquisition and control hardware. With DAQ-devices providing digital I/Os and an external power supply, any number of modules (limited by the desired dwell time and sampling rate) can be used and controlled by control- and acquisition routines written and customized by the user.

#### 2.2.2. Selection of hardware design aspects

***Emitter Branch*:** For a high accuracy of the fNIRS instrument, a careful design of the NIR-light emitting circuit is crucial, as fluctuations in the radiation intensity cannot be discriminated from changes in absorption due to changes in chromophore concentrations in the tissue.

**Figure 2:**
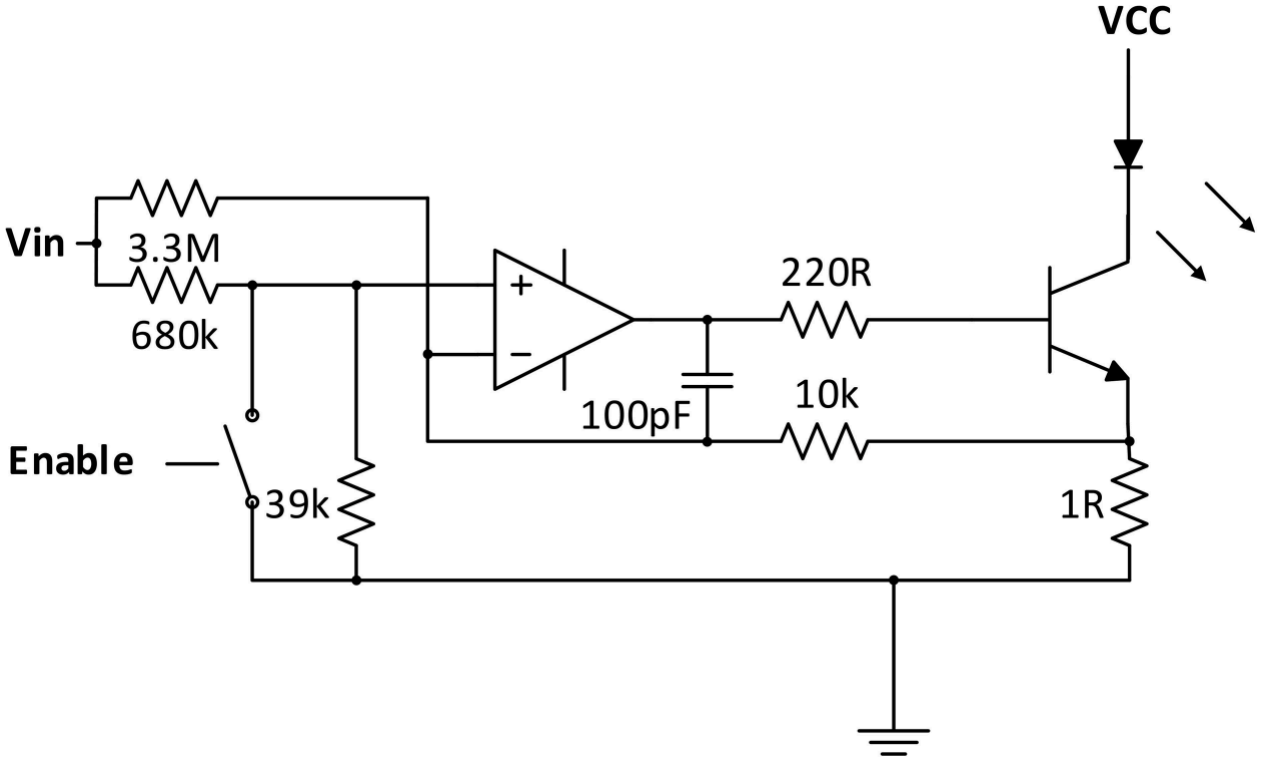
**Current regulator/modulator circuit**.

To keep the current through the LED semiconductor junctions constant and independent from variations in supply voltage and temperature, and at the same time allow intensity adjustment and current modulation for the lock-in amplification process, a customized current regulator circuit was designed (see Figure 2).

Similar to a solution proposed by Chenier and Sawan (2007), an analog switch is used in the OpAmp based regulation circuit for square-wave modulation of the current. However, instead of disrupting the regulation process at the transistor base, analog switches (ADG711) are used at the inputs of the regulator circuits to pull the regulator inputs low when deactivated. fNIRS channel activation and modulation is thus realized by simply feeding through the square wave reference to the corresponding current regulator switch selected by the multiplexer.

As the regulator is modulated in the kHz-range, over- and undershoots influence the ideally square-wave shape of the current. To optimize the shape, a passive negative RC feedback was added and evaluated for best performance.

***Receiver Branch*:** The receiver branch was designed to maximize SNR by minimizing noise influences from shot, thermal and 1∕*f* noise, dark currents and stray light from external light sources.

Shot noise is based on the quantum nature of the photons and therefore unavoidable and, for detectors without internal amplification, proportional to the square root of the average incident intensity (Scholkmann et al., 2014). To maximize SNR, the instrument is operated using the maximum NIR-light intensity level for the current regulators that is feasible in the experimental situation. Opaque cell rubber tubes are used to cover the sides of the NIR emitters and detector and the fNIRS module housing is covered with opaque paint to minimize shot noise influences from background radiation.

To reduce thermal noise influences, a Si photo diode with integrated trans-impedance amplifier circuitry (OPT101) was selected for detection. Lock-in extraction of the detected signal further reduces stray light, dark current and 1/f noise influences. Placing the PGA between the detection and lock-in extraction unit enables maximum pre-amplification of the signal while amplifier noise components added in the amplification process are reduced by the subsequent lock-in demodulation. Non-physiological high frequency components of the signal are attenuated by the 3rd order low pass filter of the lock-in demodulation unit.

#### 2.2.3. Interfaces and software design

**Figure 3:**
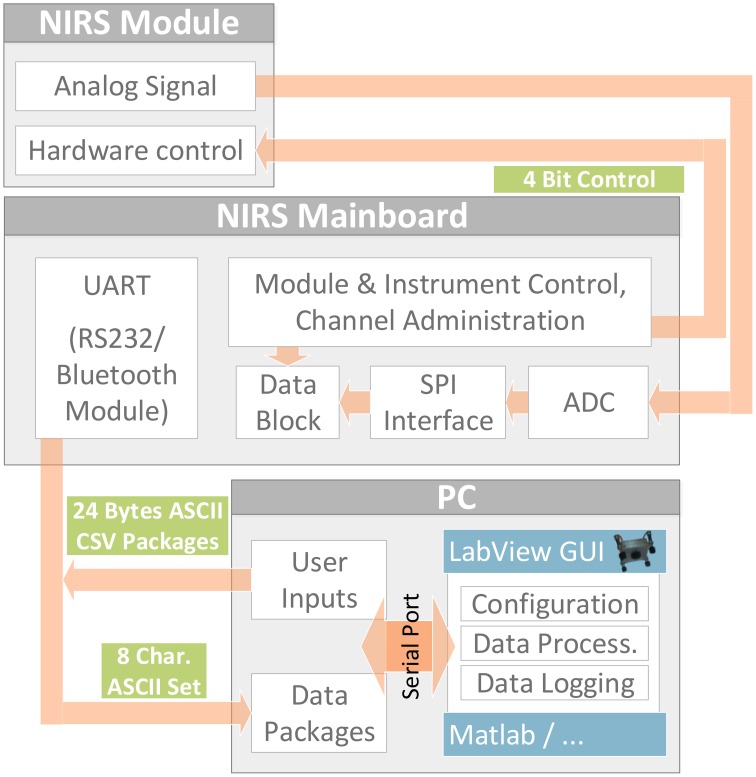
**Software and Interface Concept**. Stand alone fNIRS module operated via parallel control interface by the mainboard or any custom control and data acquisition device. Function of the 4 Bit interface (3:RST, 2:TRIG, 1:CH1, 0:CH0): Bits CH1:CH0 select one of the four physical NIRS channels. A rising edge on the TRIG line activates the selected channel, always beginning with wavelength 750 nm of the corresponding LED. Each subsequent rising edge toggles the activation between 750 and 850 nm. When the RST line is pulled up, all channels are turned off. The next rising edge on the TRIG line starts the process again, beginning with 750 nm.

Figure 3 shows the software concept. The fNIRS module software sets up hardware components (PGA, DAC, MUX,…) and is controlled by an interrupt-based architecture that receives its control signals from the 4 Bit parallel interface. Therefore, interface operation and analog signal conversion can be done by the mainboard or any custom or standard DAQ-equipment with 4Bit programmable digital outputs (such as e.g., NI USB600x series). Using the mainboard, a channel administration routine both supervises data acquisition and acts as interface between the fNIRS modules and the PC by processing received user commands (configuration, start, stop…), translating them into signals for the 4 Bit fNIRS module interface(s) and sending acquired data packages via the UART interface. On the PC's operating system side, the user can control the instrument and directly read out the data packages in ASCII CSV format via a simple serial port command console or access the serial port with any software such as LabView or Matlab. A LabView graphical user interface was developed for easy configuration and control as well as display and logging of raw and modified Beer-Lambert Law data.

#### 2.2.4. Mechanical and probe design

In the fNIRS instrument's mechanical design, the idea of modularity/scalability and robust fixation is continued by providing independent custom 3D printed solutions for the single fNIRS modules and the mainboard:

The Mainboard, Bluetooth module and batteries are worn on the upper arm of a subject in a chained multiple-unit housing (see also **Figure 5**, in the next section).

**Figure 4:**
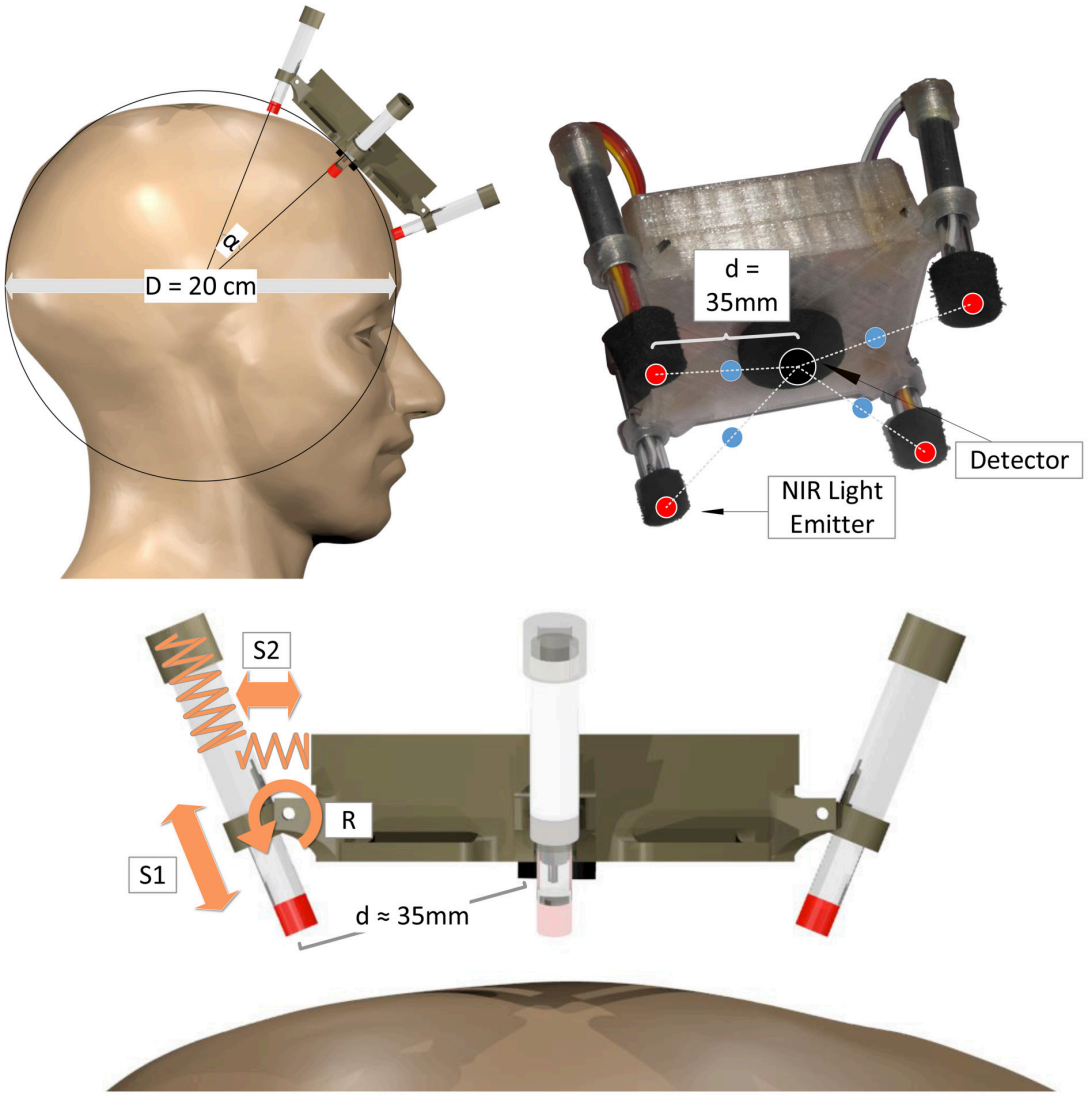
**Mechanical spring-loaded concept:** Spherical head approximation (top left), geometric channel arrangement (red: NIR LEDs, black: photo detector, blue: measurement points of highest sensitivity; top right), spring-loaded mechanical design illustrated on one LED-holder (bottom). Spring S1 for alignment and buffering, spring S2 and rotatory joint R for perpendicular alignment.

For the single fNIRS modules, a new mechanical spring-loaded design was approached to optimize signal quality, sensitivity and light penetration depth together with easy and robust, adaptive fixation of the optodes (see Figure 4). Based on a spherical approximation of the head with diameter *D* = 20 cm, the central NIR light detector and the four NIR LEDs are placed perpendicular to the scalp with a source-detector distance of *d* = 35 mm. To enable perpendicular fixation of the emitters/detector and at the same time allow alignment to the natural unevenness of the head and its deviations from the spherical approximation, the NIR light LEDs are not stiffly connected to the module body housing but integrated in movable spring-loaded LED holders. These holders are based on two nested tubes that are spring-loaded against each other (S1) and against the module housing (S2) and are able to rotate around an axis (R): Spring S1 presses the LED toward the surface of the head, thus enabling alignment and preventing the loss of contact during movements. Spring S2 and the rotary joint R keep the LED perpendicular to the surface while enabling small deviations for comfort and alignment.

To minimize stray light influences and for cushioning purposes, the detector and emitters are encased by an opaque cell rubber tubing. To fixate a single module to the head, a flexible ribbon with hook-and-loop fastener can be used that is sewed to the module housing.

The mechanical concept was designed to allow the modules to be used on the forehead as well as over haired regions of the head: The single spring-loaded optodes are easily accessible due to their modular fixation without a cap or other concealing elements. This enables the user to manually brush aside obstructing hair from under the optodes for better optical contact. Even though we successfully conducted measurements over hairy regions of the head, it has to be pointed out that the usability of the modules on other regions than the forehead has not been proven under controlled conditions so far.

### 2.3. System evaluation

#### 2.3.1. Hardware analysis

To enable a differentiated characterization of the instrument's hardware according to functional units, evaluation and analysis was split into emitter branch (current regulation and modulation), receiver branch (lock-in module), power supply stability and overall drift characteristics:

- *Current regulator/modulator speed and current shape/oscillation characteristics*: To evaluate and optimize the current regulator design characteristics for a stable and minimally oscillating but steep square wave shape of the regulated current signal, both LTSpice simulations and measurements were conducted and the regulator design parameters iteratively improved using two high-precision operational amplifiers (Analog Devices AD824A and Linear Technologies LMC6064). To minimize transient oscillation and settling times, a negative feedback decoupling capacitor *C* was introduced to the regulator design. For the determination of its optimal value, the shape of the regulated square wave current signal was investigated in a range from *C* = 0 pF to *C* = 330 pF at different current levels.
- *Lock-in performance*: The sum of propagation delays that result from each hardware component in the emitter-detector-signal path leads to an overall phase shift between input and reference signal in the analog lock-in amplification process. Such a phase shift results in an attenuation of the signal during demodulation (Meade, 1982, 1983). To minimize this effect, all hardware elements in the signal path were selected with respect to high-speed/low delay times. The remaining overall phase shift $\Delta \Phi =\frac{\Delta t}{T}\cdot 2\pi$ between the reference signal (with period *T*) and the detected pre-amplified signal was measured before demodulation. Using the established straight forward mathematical model for square wave reference lock-in demodulation, as in Meade (1983), a phase shift dependent attenuation factor
  (1) $$\begin{array}{l} A=cos(\Delta \Phi ) \end{array}$$
  was used to estimate the resulting attenuation.
  For an estimation of the receiver sensitivity using the noise equivalent power (NEP), dark voltage noise levels (no incident light to the photo detector) were measured at the output of the lock-in-module.
- *System drifts*: The following possible sources of system drift were considered: Changes in the 1Ω LED current regulation resistance due to temperature changes, changes in the total radiated power of the LEDs due to semiconductor junction temperature and changes despite constant currents and supply voltage variations. Changes in stray light, amplifier and thermal resistor noise are strongly suppressed by the lock-in amplification process. To minimize signal drifts resulting from changes in the 1Ω current regulator resistance, Panasonic current sensing resistors with a low temperature coefficient of resistance (*TCR* = ±50·10^−6^/°C) were chosen.
  The overall system drift of a single fNIRS module was specified with 20 min continuous acquisition windows of a single active channel at maximum intensity (100 mA) with the PGA set to *G* = 44 and the module being placed at a fixed position in an opaque closed box.
- *Mainboard power supply stability*: DC supply voltage drifts during 20 min signal acquisition periods and current modulation impacts on the supply voltage were evaluated. As the 100 mA (max.) square wave 3.125 kHz modulation can influence the power supply voltage stability and noise it can degrade the performance of the signal detection and amplification elements. Their output signals during active modulation were acquired while zero optical input to the photo detector was ensured by encasing the active LED with an opaque metal box. For customization, the layout of the fNIRS module allows both separate and common supply of the LED currents and module hardware.

#### 2.3.2. Physiological verification

Simple qualitative experiments were conducted using a channel at 10–20 point Fp1 to verify significant strength of physiological information in the raw signal and its power spectrum. Amongst others, visibility and strength of pulse artifacts are indicators for the signal quality and have been widely documented in fNIRS literature with the pulse artifact's amplitude being in the order of metabolic variations due to brain activity (Boas et al., 2004; Lareau et al., 2011; Scholkmann et al., 2014). Thus, with the fNIRS module pressed firmly against the head to reduce the sensitivity to scalp signals (decreased blood flow under the optodes), a clearly visible pulse artifact is a first indicator for sufficient signal quality to measure brain activation. The pulse rate was verified with conventional reference pulse measurements.

For verification and quantification of the device's capability to measure metabolic brain activity, a mental arithmetic BCI experiment was conducted with 12 subjects. In this experiment, it is shown that the measured hemodynamic responses can be classified on a single-trial basis, i.e., each trial can be classified as containing mental arithmetic or relaxation, instead of measuring only the difference in the average hemodynamic response.

Mental arithmetic tasks are known to illicit strong hemodynamic reactions in frontal brain areas and have been investigated in a variate of studies with fNIRS (Ang et al., 2010; Herff et al., 2013; Bauernfeind et al., 2014). Here, 30 trials of mental arithmetic data were recorded for each participant. During each 10 s trial, participants were asked to repeatedly subtract a number between 7 and 19 (excluding 10) from a number between 501 and 999). Both numbers were presented on a screen at a distance of roughly 50 cm. After each mental arithmetic trial, participants were asked to relax for 25–30 s. These pause intervals were indicated by a fixation cross on the screen. A longer resting period of variable length was included after 15 trials to allow participants to rest and drink. No data of these extended resting periods were used in our analysis.

The open fNIRS device was placed on the forehead and fixated around the head with the flexible ribbon with hook-and-loop fastener sewed to its housing. It was placed such that both active emitters were placed on the locations Fp1 and Fp2 of the international 10–20-system. The light detector was placed on AFz resulting in an emitter-detector distance of approximately 3.5 cm.

All subjects were informed prior to the experiment and gave written consent.

The signal processing of the recorded data was performed in a straight-forward and simple manner, since we focus on the developed hardware in this paper. More advanced methods have been shown to improve accuracies for classification in neuroimaging (Calhoun et al., 2001; Blankertz et al., 2008; Lemm et al., 2011; Heger et al., 2014). The raw optical densities were transferred to concentration changes of oxygenated and deoxygenated hemoglobin (HbO and HbR, respectively) using the modified Beer-Lambert Law (Sassaroli and Fantini, 2004). HbO and HbR values were then linearly detrended in windows of 300 s. Low frequency noise was attenuated by subtracting a moving average of the mean of 30 s prior and after every sample. Finally the data was low-pass filtered using an elliptic IIR filter with filter order 6 and a cut-off frequency of 0.5 Hz to reduce high-frequency systemic noise like pulse artifacts.

After preprocessing, trials were extracted based on the experiment timings. For the pause blocks, we extracted the last 10 s of the 25–30 s pause intervals, to ensure that hemoglobin levels have returned to baseline. For each mental arithmetic trial, we extracted 10 s of data starting 5 s after stimulus presentation, to ensure that the hemodynamic response has already developed. Labels were assigned to the trials referring to either mental arithmetics or pause data. For each trial, we extracted the slope of a straight line fitted to the HbO and HbR data of each channel as a feature. The line was fitted using linear regression with a least-squares approach. Slope features have been shown to work well in previous studies (Herff et al., 2014).

Evaluation was performed using a 10-fold cross-validation and classification by Linear Discriminant Analysis. In addition to the single trial analysis, the average hemodynamic response is calculated by averaging over all mental arithmetics or all pause trials.

## 3. Results

**Figure 5:**
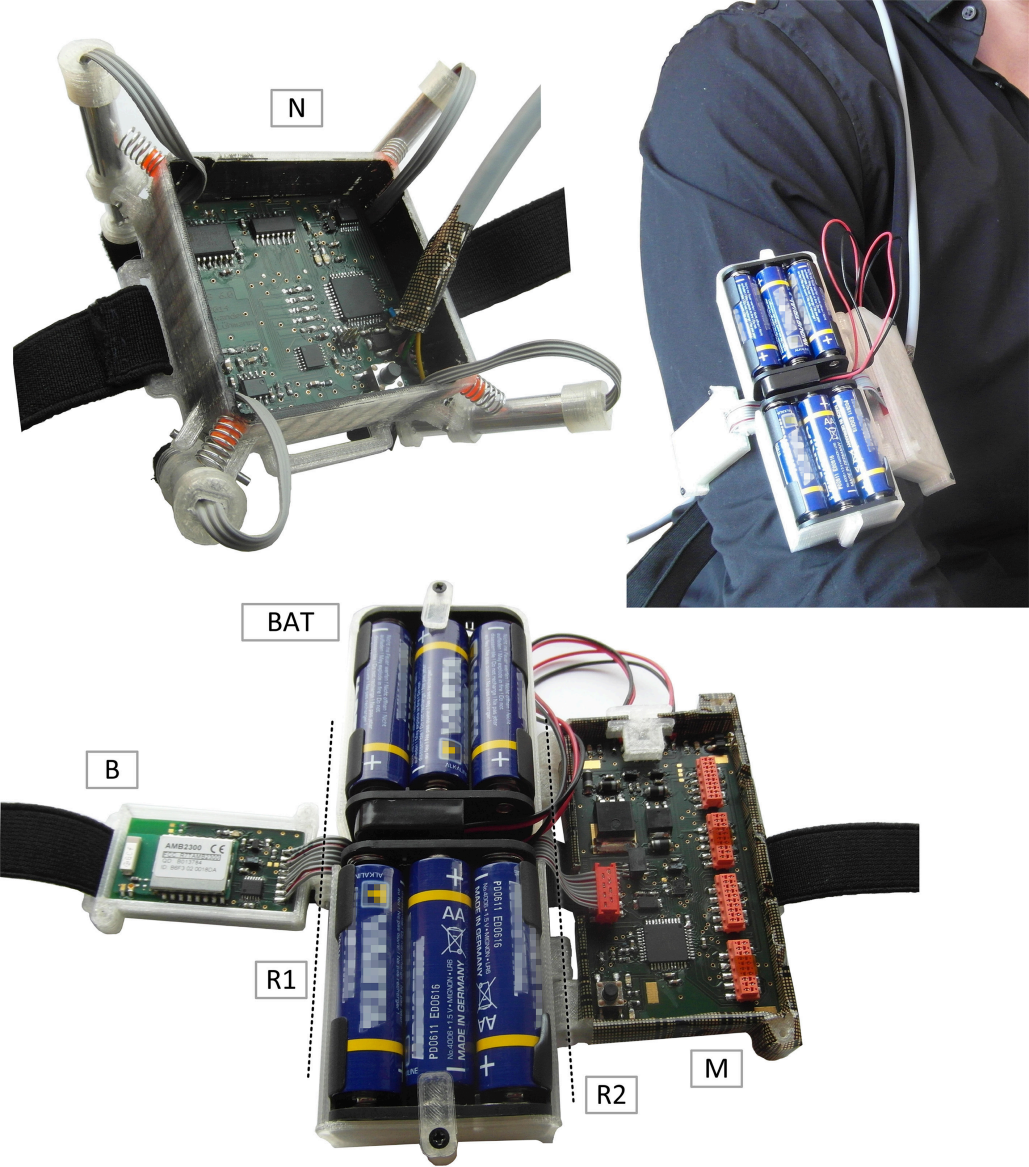
**Final System**. (N): single 4 channel fNIRS module. Top right/bottom: Chained mainboard module. B, bluetooth module; BAT, batteries; R1/R2, Rotatory joints; M, mainboard.

The developed open modular multichannel fNIRS system (see Figure 5) proved functionality, fast set up and easy application in all testing conditions. Wearing the mainboard module on the upper arm and the fNIRS module on the head using flexible ribbons and hook-and-loop fastener, the user can move freely and is bothered minimally by the instrument while signal quality and robustness to movement showed promising results. It should be noted however, that the physiological signals used for evaluation results in this paper were acquired from sitting subjects to reduce possible error sources and thus allow a more explicit first performance assessment of the new open source hardware.

The final instrument is characterized by:

- Modularity, customization and stand-alone functionality.
- Optimized adjustable current regulation and modulation with negative decoupling.
- A lock-in-based signal extraction module with programmable amplification.
- A 4-channel spring-loaded mechanical concept for fNIRS probe attachment to improve user comfort and robustness against movement artifacts.
- A microcontroller and Bluetooth based mainboard as interchangeable peripheral control, acquisition and transmission hardware with a Bluetooth range of max. 20 m (optimal open field conditions).

Low cost components were used for the design. The total cost of the instrument's hardware for one 4 channel fNIRS module and one mainboard mainly depends on PCB fabrication costs and is approximately 200*EUR*∕250*USD*.

A full documentation including detailed descriptions, schematics, and evaluation can be found in the supplementary materials for this article/on the web: www.opennirs.org. In the following, we present the main evaluation results of the steps described in section 2.

### 3.1. Current regulation/modulation circuit

**Figure 6:**
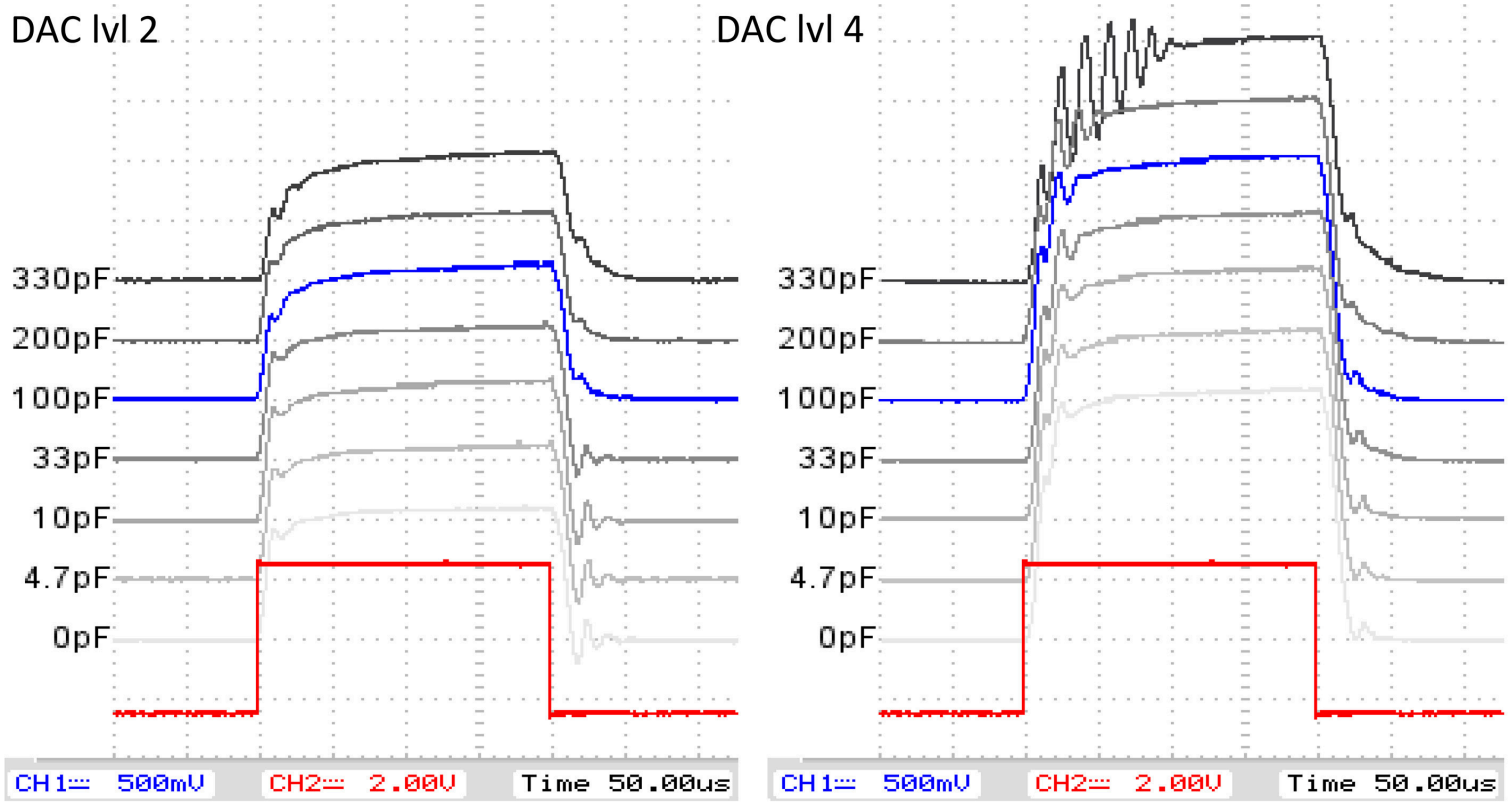
**Current regulator/moulator evaluation:** 3.125 kHz PWM modulation reference (red) and regulated LED currents with different decoupling capacitor values and DAC levels (blue: selected value for design).

Mostly due to its higher slew rate, the AD824A showed a much faster current regulation and lower transient oscillations than the LMC6064. The experimental results for the minimization of oscillation and settling times with different decoupling capacitor values *C* (see Figure 6) showed higher settling oscillations for low *C* at low current levels, higher transient oscillation for high *C* at higher current levels and allowed the identification of the optimal *C*: *C* = 100 pF showed the best tradeoff between minimal oscillations and maximal edge steepness for all current levels.

### 3.2. System drifts

The signal drift of a continuously active channel in Volts per second was calculated using linear least squares regression on the acquired 20 min. raw signals, yielding a negative drift coefficient of $C_{D}=-1\cdot 10^{-6}$ V/s (measured fNIRS signals typically—dependent on the device configuration—being in the order of several hundred mV) and a respective long term stability coefficient of < −0.42% for both wavelengths. It was observed that independent from the fNIRS module, power supply heating on the peripheral hardware can add additional drifts of up to one order of magnitude by effecting the analog-to-digital converter. This points out the importance of careful selection/design of peripheral hardware for the acquisition of the fNIRS module's analog signal.

### 3.3. Lock-in amplification, SNR and dynamic range

For an approximation of the total effective phase shift between reference and demodulator input signal in the lock-in unit, the delays between both signals were measured at the 50% levels of both respective rising (*t*_*dr*_) and falling edges (*t*_*df*_). It is the sum of times where the logical levels of both signals do not match and was measured as Δ*t* = *t*_*dr*_+*t*_*df*_ = 18.5+7.2 μs. To estimate the attenuation caused by non-phase-synchronous demodulation of the signal A, Equation (1) is used with the measured Δ*t* and reference signal cycle duration *T* = 320 μs, and yields *A*≈0.875, which does not affect the overall accuracy significantly. Evaluation of the single component phase delays in the signal path revealed that further minimization approaches should first target the PGA (Δ*t*_*PGA*_ = 7.0+4.5 μs).

For the evaluation of the detector's sensitivity and dynamic range, the mean dark voltage signal μ_*d*_ (no incident light on the photodetector) at the output of the lock-in amplifier and post amplification branch was measured to be μ_*d*_ = 0.101 Vrms with a standard deviation of σ_*d*_ = 3.99 mVrms at a typical PGA gain of *G* = 44 and fixed lock-in filter gain of *G* = 5.1. Using the mean dark voltage plus standard deviation and the responsivities *R*_λ_ of the OPT101 photodiode (*R*_750_ = 0.55 V/μW and *R*_850_ = 0.60 V/μW), the Noise Equivalent Powers of the whole detector branch for a SNR of one

(2) $$\begin{array}{l} NEP_{\lambda }=\frac{\mu_{d}+\sigma_{d}}{R_{\lambda }\cdot G_{total}} \end{array}$$

were estimated to be *NEP*_750_ = 2.27 nWpp = 0.80 nWrms and *NEP*_850_ = 2.21 nWpp = 0.78 nWrms.

**Figure 7:**
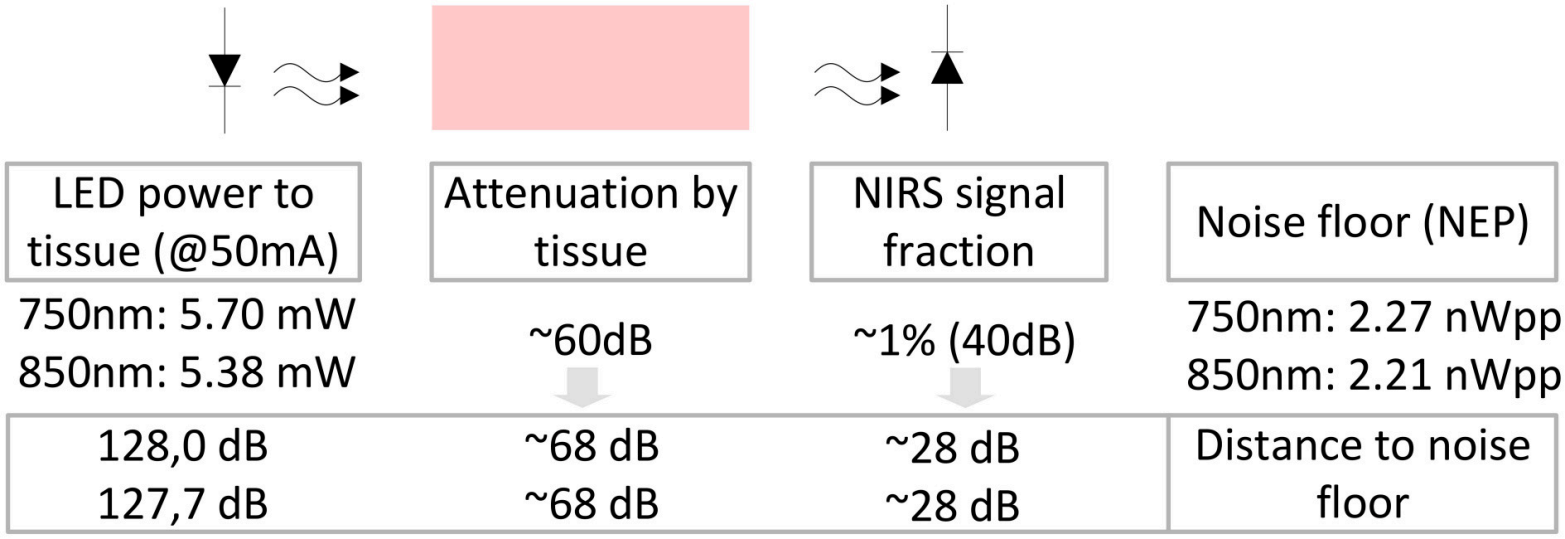
**Estimation of signal and noise in the instrument:** With the NEPs identified to be 2.27/2.21 nWpp, the distance between these optical powers equivalent to the noise floor of the detection circuit and the measured powers incident to the tissue (5.70 mW/5.38 mW) at medium LED illumination can be determined to be approximately 128 dB/127.7 dB, respectively. The degree of optical loss of the incident light in the tissue is subject dependent. Here, we estimate it to be in the order of at least 40–60 dB. With the actual metabolic fNIRS signal being in the order of 1% of the measured optical signal, the distance of the fNIRS signals to the noise floor is approximately 28 dB.

The optical powers radiated by the LED at medium intensity (*I*_*F*_ = 50 mA) were measured to be 5.70 mW for 750 nm and 5.38 mW for 850 nm. Using these incident powers and the NEPs allows an estimation of the signal to noise distances (for an overview see Figure 7): The wavelength dependent ratio of incident light to light not longer detectable as its signal is drowning in noise, yields signal to noise distances of 128 dB_750_ and 127.7 dB_850_. These distances are largely decreased by the optical loss due to tissue scattering and absorption that is subject dependent and assumed to be in the order of >60 dB. With the physiological fNIRS signal usually being around 1% of the measured optical signal, the distance between the fNIRS signal components and the noise floor of the detection circuit is further decreased by ≈40 dB and estimated to be in the order of 28 dB.

Saturation of the detection branch occurs, when the upper input voltage limit of the ADC, here 2.5 Vpp, is reached for the lowest PGA gain setting of *G* = 0.6875, which is the case at 1.296∕1.188 μWpp incident light (750 nm/850 nm). Using these results, the minimum system dynamic range, expressed as the ratio of signal saturation to the NEPs, is estimated to be in the order of 55.13∕54.6 dB. It should be stated, that the configuration of the LED intensities (25–100 mA) on the emitter side can further increase the dynamic range of the instrument. Table 1 summarizes the performance characteristics.

**Table 1:** **Performance characteristics of the fNIRS Module**.

**Parameter**	**Value**
OPT101 Dark noise voltage	300 μVrms @0.1–20 kHz
OPT101 Responsivity	*R*_750_≈0.55 V/μW
	*R*_850_≈0.60 V/μW
Noise equivalent power	*NEP*_750_≈0.80 nWrms
	*NEP*_850_≈0.78 nWrms
Power to tissue (at peak wavelength)	
@*I*_*LED*_ = 50 mA	*P*_750_ = 5.70 mW
	*P*_850_ = 5.38 mW
@*I*_*LED*_ = 100 mA	*P*_750_ = 11.10 mW
	*P*_850_ = 10.30 mW
Est. signal to noise distance	
@*I*_*LED*_ = 50 mA	≈28 dB
Eff. dynamic range	>55 dB
Signal drift	< −1·10^−6^ V/s/ < −0.5%
Sampling rate	Variable, dwell time dependent

### 3.4. Mainboard power supply

The ±5 V DC supply voltage drift measurements showed a stable supply voltage of +4.959 V and −4.960 V with less than 500 μV total drift in 20-min measurement periods. Evaluation of the maximum impact of the current modulation on the detecting components via the power supply revealed that current modulation flanks can create a ±2 mV high-frequency(kHz) noise around the photo detector output baseline signal that is further amplified by the PGA to strong ±100 mV peaks (at *G* = 44) when supplying the LED current either directly from the battery or from the regulated +5 V rail for the other fNIRS module hardware. However, as the supply variations are synchronous with the signals and as high-frequency noise is effectively suppressed by the 3rd-order lock-in low-pass of the fNIRS module, influences on the baseline of the lock-in demodulated signal were not observed.

### 3.5. Physiological measurements

**Figure 8:**
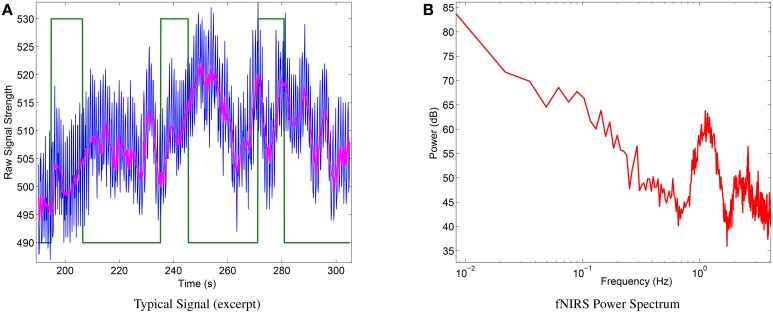
**(A)** Excerpt of typical raw signal (blue) during mental arithmetics. Green line: binary label (high states: m. arithmetics. low states: relax), magenta line: median filtered signal). **(B)** Typical power spectrum of raw signal of the same subject and complete session, showing expected shape (power spectrum follows power law) and deviations caused by systemic artifacts.

Qualitative physiological experiments showed very clear signals and proved the basic functionality of the instrument. Figure 8 shows a representative raw signal (750 nm) during three mental arithmetics trials performed by a subject (a) and the power spectrum computed over the whole session for the same subject (b). The latter shows the typical power law appearance and peaks by systemic artifacts that have widely been reported for fNIRS signals in the literature (e.g., Fekete et al., 2011).

**Figure 9:**
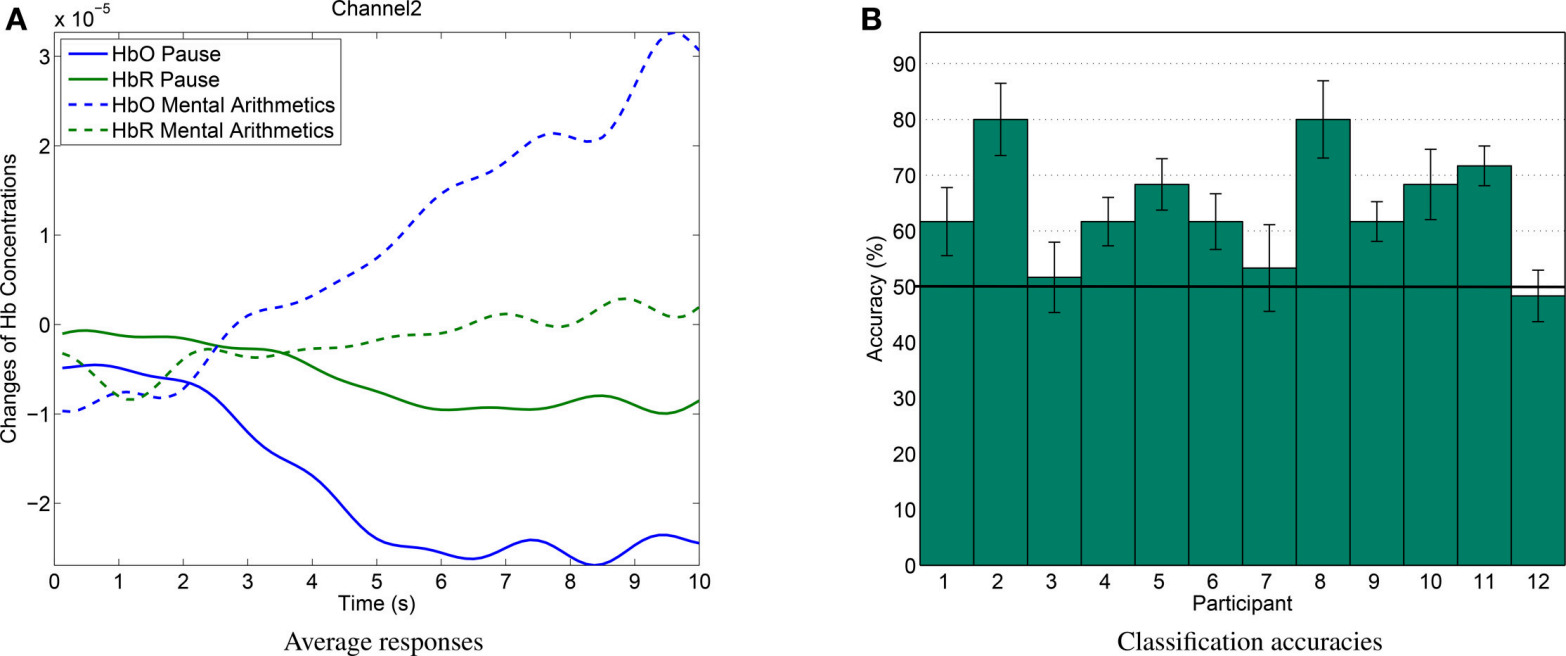
**(A)** Average hemodynamic response over all subjects during mental arithmetics and pause. **(B)** Classification results for single-trial discrimination between pause and mental arithmetics. Whiskers indicate standard errors. Solid line shows chance level.

The average hemodynamic response (see Figure 9A) over all subjects of the mental arithmetics experiment shows the expected behavior, i.e., an increase in HbO peaking after approximately 10 s during mental arithmetics. During the average pause interval, HbO levels still slowly return to baseline after the preceding activation.

Discrimination between pause and mental arithmetics yielded an average of 65.14% accuracy. Of the 12 recorded participants, 9 yielded accuracies significantly higher than chance level (one-sided *t*-test, *p* < 0.05). Classification results for all participants can be seen in Figure 9B). In a similar study by Herff et al. (2013), mental arithmetics could be discriminated from pause with 71.17% using 8 channels and 67.26% when using only two channels at similar positions as in this study.

## 4. Discussion and conclusion

### 4.1. Key findings

In the beginning of this paper, we identified system requirements for mobile fNIRS based neuroergonomics/BCI applications. The results indicate, that the presented open source device satisfies the requirements.

In the course of the experiments, both, experimentators and subjects, appraised the usability of the device to be high. Miniaturization of the modules and mobility through Bluetooth based wireless transmission allowed free movement, the use of commercial reference systems usually required longer preparation times for optode fixation and was often uncomfortable and static because of the weight of the optical fiber guides and the lack of cushioning of the optodes. In contrast, the new wearable system could be applied within several seconds and was generally perceived less cumbersome during the experiments.

The hardware evaluation results and physiological verification of the designed miniaturized fNIRS instrument indicated a sufficient signal quality and system performance for brain activity measurements with an approximated signal to noise distance of 28 dB. The lock-in amplifier, detector sensitivity, current modulation precision and drift evaluation of the device showed satisfying results comparable to other documented fNIRS devices. The physiological measurements showed the expected hemodynamic responses, classification accuracies in single-trial analysis exceeded chance level for 9 out of 12 participants and yielded results comparable to those measured with a commercial device in a similar study (Herff et al., 2013) using 2 of 8 channels at similar positions (65.14 vs. 67.26%). The open fNIRS device can thus be used for mobile fNIRS-based BCI and neuroergonomics applications.

Battery supply and wireless communication, low heating due to time multiplexing of the channels and the use of LEDs as light sources assured a safe usage of the device.

The scalable modular concept, configurable light intensities and detector amplification gains and the flexible parallel interface of the fNIRS modules allow easy customization and configuration of the hardware.

However, there are still several elements in the design that can be optimized to further improve instrument performance in the future.

### 4.2. Limitations and next steps

#### 4.2.1. Mainboard/data acquisition and control

An obvious but crucial component for the use of the fNIRS module is the data acquisition unit. When using custom hardware for data acquisition, the design and selection of the analog-to-digital converter (ADC) determine not only quantization depth but also the frequency resolution of the time division multiplexed fNIRS channels, as the ADC sampling rate has to be shared by the up to 4 active channels of one module. The ADC (LTC2486) first used on the mainboard offered 16 Bit conversion depth and exceptional DC accuracy but significantly limited time resolution due to a conversion time of type 80.3 ms. Additional experiments indicated that, using ADCs with significantly higher sample rate but lower resolution, down to 10 Bit quantization depth can suffice for reliable brain activation measurements. Future designs of the mainboard/DAQ hardware should therefore aim to use a better suited (faster) ADC to prevent the sampling frequency bottleneck. Here, the modular concept is advantageous, as the DAQ-unit can be customized and optimized independent from the hardware of the fNIRS modules.

Power supply and current modulation impact evaluation showed, that even though the implemented linear-voltage-regulator-based symmetric supply appeared to be sufficient, several improvements can be suggested for use with the fNIRS module:

To minimize crosstalk between the modulated NIR-LED current and the regulated ±5 V supply voltage rail for the detection hardware, supplying the LEDs with a separate additional voltage regulator circuit is preferable over the use of a common regulator or direct battery connection in the design. Implementation of additional high-frequency filters and enhanced stabilization are also recommended in future approaches to reduce noise pickup from external sources and further minimize LED current modulation influences on the rest of the system. The use of voltage regulators with higher efficiency can further enhance battery life and decrease heating effects, which also can—dependent on the supplying and acquisition hardware's design and layout—influence system drifts.

#### 4.2.2. fNIRS module

The phase delay dependent attenuation of approximately 0.875 by the lock-in detector is acceptable as it does not significantly decrease overall system accuracy. However, it can be further minimized: To improve the lock-in performance, an analog adjustment of the PWM reference phase could be implemented for overall phase shift compensation. Alternatively, a potentially superior approach for a next-generation design would be digital lock-in demodulation based on a microcontroller/DSP. This bears several advantages: reduced cost of hardware components, reduced power consumption and an adjustable phase shift correction and thus higher precision.

The four channel set up per module using four LEDs and one photodiode was necessary for this first approach using a single-channel analog lock-in receiver branch for a simple interface in favor of modularity. However, to further reduce energy consumption and increase channel density, future approaches should utilize configurations with more PDs measuring simultaneously. Additionally, although the fNIRS module is already compact and provides stand-alone functionality, further miniaturization is possible. A next step will be the development of entirely stand-alone modules to redundantize peripheral hardware such as the mainboard. Integrating the above mentioned insights and data acquisition, digital lock in, power management and wireless transmission components onto a further miniaturized multichannel fNIRS module could enable even more applications in and out of the lab.

The instrument can be improved and evaluated in several more ways. However, providing this fNIRS device open source, we hope that aspects of this work will be helpful to further simplify and reduce time and effort in future custom fNIRS based mobile BCI and neuroergonomics approaches.

### Conflict of interest statement

The Review Editor Laurens Ruben Krol declares that, despite being affiliated with the same institution as the Author Alexander Von Lühmann, the review process was handled objectively. The authors declare that the research was conducted in the absence of any commercial or financial relationships that could be construed as a potential conflict of interest.
